# Evaluation of Host-Derived Volatiles for Trapping *Culicoides* Biting Midges (Diptera: Ceratopogonidae)

**DOI:** 10.1007/s10886-017-0860-x

**Published:** 2017-07-03

**Authors:** Elin Isberg, Daniel Peter Bray, Ylva Hillbur, Rickard Ignell

**Affiliations:** 1 Unit of Chemical Ecology, Department of Plant Protection Biology Swedish University of Agricultural Sciences, Box 102, 230 53 Alnarp, Sweden; 2 Agriculture, Health and Environment Department, Natural Resources Institute, University of Greenwich, London, ME4 4TB UK; 3 International Institute for Tropical Agriculture, Oyo Road, Ibadan, Nigeria

**Keywords:** Attractants, Biting midges, Vectors, Arbovirus, Carbon dioxide, Host-odours, Kairomones, Repellents

## Abstract

*Culicoides* biting midges (Diptera: Ceratopognidae) cause pain and distress through blood feeding, and transmit viruses that threaten both animal and human health worldwide. There are few effective tools for monitoring and control of biting midges, with semiochemical-based strategies offering the advantage of targeting host-seeking populations. In previous studies, we identified the host preference of multiple *Culicoides* species, including *Culicoides impunctatus*, as well as cattle-derived compounds that modulate the behavioral responses of *C. nubeculosus* under laboratory conditions. Here, we test the efficacy of these compounds, when released at different rates, in attracting *C. impunctatus* under field conditions in Southern Sweden. Traps releasing 1-octen-3-ol, decanal, phenol, 4-methylphenol or 3-propylphenol, when combined with carbon dioxide (CO_2_), captured significantly higher numbers of *C. impunctatus* compared to control traps baited with CO_2_ alone, with low release rates (0.1 mg h^−1^, 1 mg h^−1^) being generally more attractive. In contrast, traps releasing octanal or (*E*)-2-nonenal at 1 mg h^−1^ and 10 mg h^−1^ collected significantly lower numbers of *C. impunctatus* than control traps baited with CO_2_ only. Nonanal and 2-ethylhexanol did not affect the attraction of *C. impunctatus* when compared to CO_2_ alone at any of the release rates tested. The potential use of these semiochemicals as attractants and repellents for biting midge control is discussed.

## Introduction

*Culicoides* biting midges (Diptera: Ceratopognidae) are vectors of viruses of both medical and veterinary importance (Purse and Venter 2015). African horse sickness virus (AHSV), epizootic haemorrhagic disease virus (EHDV) and bluetongue virus (BTV) are listed by the Office International des Epizooties as posing a high risk to animal health where competent vectors are present. The emergence of Schmallenberg disease in Europe has further highlighted the importance of these insects in transmitting new zoonoses endangering animal welfare (Carpenter et al. 2013). Even in the absence of disease, the pain and distress of *Culicoides* blood feeding causes major economic losses (Mordue and Mordue 2003), and can result in life-threatening allergic reactions (Carpenter et al. 2008a).

*Culicoides impunctatus* is geographically one of the most widespread pest species of biting midge across the West Palaearctic region (Mathieu et al. 2012). The species has a broad host range, including both wildlife and livestock (Blackwell et al. 1995), and causes economic losses to tourism and forestry through voracious blood feeding on people (Hendry and Godwin 1988). Female *C. impunctatus* are autogenous, producing one batch of eggs prior to a blood meal, facilitating population growth up to huge densities even where hosts are not readily available (Blackwell et al. 1992; Boorman and Goddard 1970). In addition to being a serious economic pest, *C. impunctatus* is also susceptible to infection by BTV, as determined under laboratory conditions (Carpenter et al. 2006). Given the high population densities and biting rates that can be attained by *C. impunctatus*, the potential of this species to act as a vector of disease cannot be entirely discounted (Purse et al. 2012).

Currently there are few effective tools for the surveillance and control of biting midge populations (Carpenter et al. 2008a). Traps baited with carbon dioxide (CO_2_) and 1-octen-3-ol, kairomones emitted by mammalian hosts, have been tested and marketed for controlling populations of biting midge, including *C. impunctatus* (Mands et al. 2004). While successful in capturing biting midges, these traps were originally designed for catching mosquitoes, and may not be fully optimized for use against *C. impunctatus* and other biting midge species. Laboratory and field studies have demonstrated that *C. impunctatus* responds to a wide range of host-produced kairomones, including acetone, butanone, lactic acid and a number of phenolic compounds (Bhasin et al. 2000; Logan et al. 2009). The addition of cow urine and hexane extracts of hair samples from large animals have also been shown to increase the attraction of *C. impunctatus* to traps baited with CO_2_ alone, and CO_2_ and 1-octen-3-ol, respectively (Bhasin et al. 2001; Mands et al. 2004). While the chemical components responsible for this increase in attraction of *C. impunctatus* to animal odor have not been elucidated fully, in analyses by coupled gas chromatography and electroantennographic detection (GC-EAD) in our laboratory volatile components have been identified from cow urine and hair that elicit antennal responses in a related species, *C. nubeculosus* (Isberg et al. 2016). In a laboratory behavioral assay, 1-octen-3-ol, decanal, 2-ethylhexanol, phenol and 4-methylphenol elicited attraction of *C. nubeculosus* when combined with CO_2_, whereas octanal, nonanal, (*E*)-2-nonenal and 3-propylphenol acted as behavioral inhibitors. The behavioral effect of these volatiles was dose dependent.

The aim of this study was to determine the release rates of the compounds identified from cow urine and hair that either increase or reduce the attraction of *C. impunctatus* to traps baited with CO_2_-in the field. The experiments were conducted in Southern Sweden, an area where livestock are at risk to midge-borne diseases (Doréa et al. 2016; Hultén et al. 2013), and where biting midge populations feed voraciously on people and animals during the period between April to October (Ander et al. 2012). We discuss our findings in relation to their potential use in future surveillance and control strategies targeting biting midges.

## Methods and Materials

### Study Site

Field trapping was performed at Stockhultsgården, 14 km northwest of Markaryd, Sweden (N 56° 32,867′, E 13° 32.542′), from June to mid-July, the peak season for blood-feeding *C. impunctatus* in Southern Sweden. The field site, a meadow (approximately 2.6 ha), surrounded by a mixture of evergreen and deciduous trees, sustains a large population of *C. impunctatus*. As a well-established hunting ground, the area is inhabited by populations of elk, fallow deer, roe deer and wild boar, all potential hosts of biting midges (Blackwell et al. 1994; Pettersson et al. 2012; Viennet et al. 2013).

### Odour Compounds

The compounds tested in this study have previously been identified in aerations of cattle hair and urine, and shown to elicit electrophysiological and behavioral responses in *C. nubeculosus* in the laboratory (Isberg et al. 2016). Chemicals used were sourced from Sigma-Aldrich Chemie GmbH, Steinheim, Germany, except for 3-propylphenol which was obtained from Alfa Aesar GmbH Karlsruhe, Germany (Table 1).

**Table 1:** Odor compounds and dispensers used in combination with carbon dioxide in field tests of trapping *Culicoides* biting midges and target release rates

Compound	Purity (%)	Origin	Release rate					
			approx 0.1 mg h^−1^		approx 1 mg h^−1^		approx 10 mg h^−1^	
			No. vials	Hole dia (mm)	No. vials	Hole dia (mm)	No.vials	Hole dia (mm)
Octanal	98	Cattle hair	1	4	2	6	2	16
Nonanal	95	Cattle hair	1	8	2	10	6	16
Decanal	98	Cattle hair	1	8	1	16	9	16
(*E*)-2-nonenal	97	Cattle hair	1	6	1	16	9	16
2-Ethylhexanol	99	Cattle urine	1	6	2	10	9	16
1-Octen-3-ol	98	Cattle hair	1	4	1	10	5	16
Phenol	99	Cattle urine	1	6	1	10	5	open^a^
4-Methylphenol	99	Cattle urine	1	6	2	10	6	open^a^
3-Propylphenol	98	Cattle urine	1	8	2	10	10	open^a^

Dispensers were polyethylene vials (height 32.9 mm, diameter 22.8 mm) with a hole drilled in the lid

^a^Lid removed from vial

### Determination of Release Rates

Test compounds (100 μl) were released via holes drilled in the lids of polyethylene vials (Kartell Labware, Noviglio, Italy; height 32.9 mm, diameter 22.8 mm, wall thickness 1.35 mm) at three different approximate release rates of 0.1 mg h^−1^, 1 mg h^−1^ and 10 mg h^−1^. The size of the holes and number of vials required for each combination of compound and release rate (Table 1) were determined through preliminary experiments. Vials were weighed prior to and following 24 h of outdoor exposure, with weights recorded every hour for the first 6 h. This procedure was repeated twice, using two vials per compound per repetition, to obtain an average release rate for each compound to be used in trapping experiments (Table 2). Maximum and minimum temperatures during the measurement period were 19 °C and 8 °C respectively, similar to those during the period of trapping experiments (16 °C and 9 °C respectively).

**Table 2:** Average release rates (± SEM) over 24 h of odor compounds from vials with lids with different sized holes (*N* = 4; 8 °C – 19 °C)

Compound	Lid hole diameter (mm)	Release rate (mg h^−1^)
Octanal	4	0.18 ± 0.07
	6	0.48 ± 0.27
	16	5.47 ± 0.38
Nonanal	8	0.12 ± 0.03
	10	0.50 ± 0.14
	16	1.62 ± 0.18
Decanal	8	0.08 ± 0.12
	16	1.12 ± 0.03
(*E*)-2-nonenal	6	0.18 ± 0.14
	16	1.15 ± 0.11
2-Ethylhexanol	6	0.20 ± 0.09
	10	0.43 ± 0.17
	16	1.12 ± 0.17
1-Octen-3-ol	4	0.18 ± 0.10
	10	1.05 ± 0.07
	16	2.05 ± 0.48
Phenol	6	0.15 ± 0.03
	10	0.93 ± 0.28
	open	1.92 ± 0.34
4-Methylphenol	6	0.10 ± 0.08
	10	0.60 ± 0.15
	open	1.58 ± 0.38
3-Propylphenol	8	0.15 ± 0.10
	10	0.48 ± 0.20
	open	0.93 ± 0.20

### Trapping Protocol

**Fig. 1:**
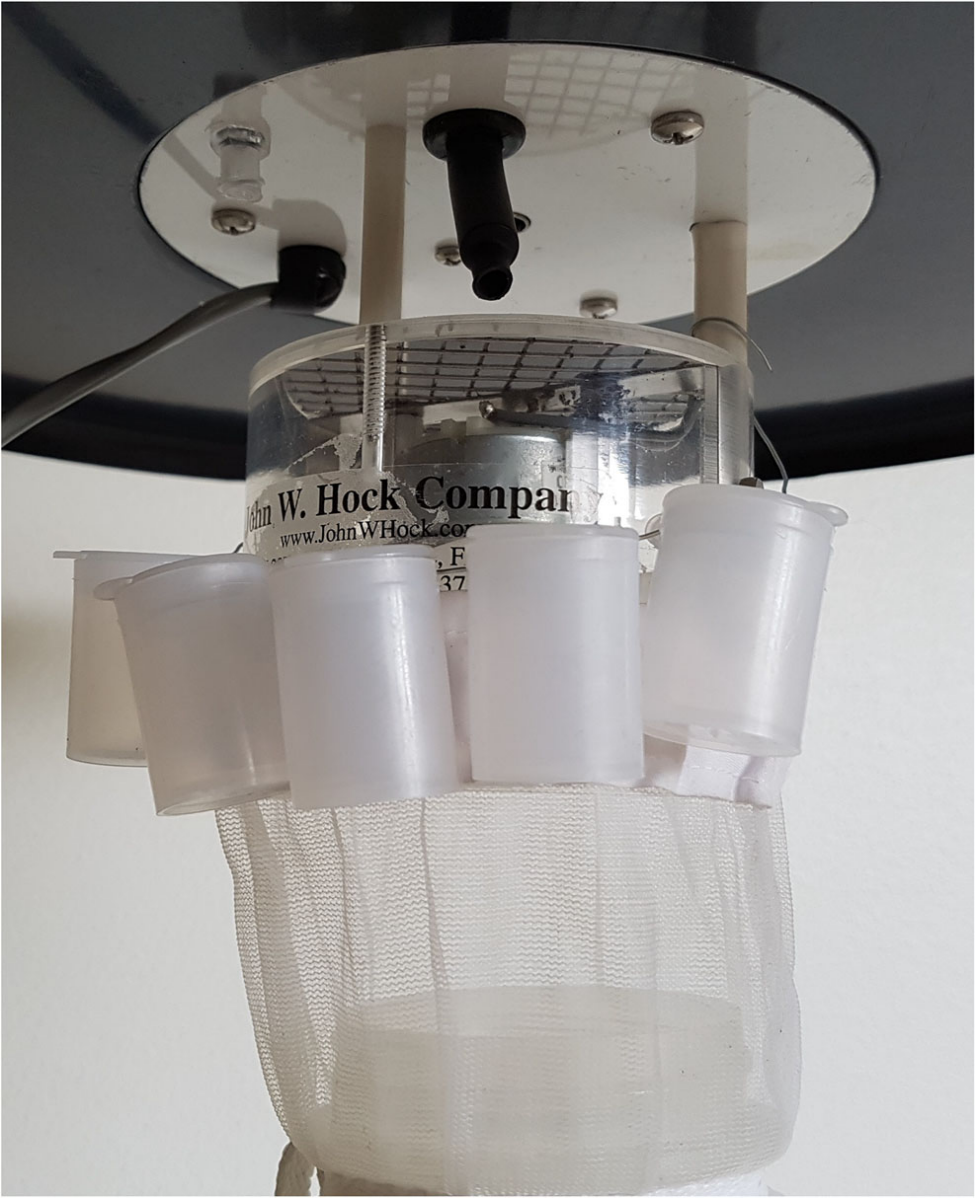
Placement of vials releasing odor compounds on the CDC light trap

The field site was divided into five sub-sites, each separated by at least 100 m. To avoid cross contamination, only one compound was tested at each sub-site at any one time, and each compound was only tested at one sub-site. Traps used were Centers for Disease Control and Prevention (CDC) standard miniature light traps fitted with a CO_2_ delivery system (Model 1012-CO_2_; The John W. Hock Company, Gainesville, Florida). Carbon dioxide from a cylinder (Strandmöllen AB, Ljungby, Sweden) was released at 500 ml min^−1^ (Bhasin et al. 2001; Harrup et al. 2012) from both control and test traps. The vials containing test compounds were hung on the outside of the trap close to the CO_2_-release point of the test traps (Fig. 1).

A randomized design was used to study the individual odor compounds at different release rates. One control trap, releasing CO_2_ only, was placed with the three traps baited with the individual odor compounds released at different rates, together forming a test group. One test group was placed at one of the sub-sites in the form of a square, with traps 1.5 m from the ground, 3 m apart. This experimental set up was selected to counteract high temporal and spatial variation of biting midge densities that can increase or decrease dramatically over distances less than 50 m (Kirkeby et al. 2013; personal observations). The positions of the traps were randomly placed within the test group before every test night, using a computerized randomization scheme (Minitab® 15 Statistical Software, Minitab Inc. UK).

Each trap was set to operate from 2 h before sunset until 1 h after sunset, for a total of seven nights, with vials replaced after every night. Insects attracted to each trap were drawn into a collection bottle containing a dilute detergent solution. Bottles were emptied daily, and the insects placed in 75% ethanol for storage and transportation. In the laboratory, the numbers of biting midges collected were recorded and identified to species level under a microscope using a morphological identification key (Delecolle 1985). Larger samples, >1000 biting midges, were subsampled according to the method of Vanark and Meiswinkel (1992) to obtain an estimate of the total number of biting midges in the sample.

### Statistical Analysis

The goal of the statistical analyses was to identify release rates for each compound, which, when used in combination with CO_2_, attracted significantly higher or lower numbers of biting midges than the CO_2_-only control. A mixed modelling approach was used to control for variation in numbers of biting midges collected between days (Paterson and Lello 2003), using the lme4 (Bates et al. 2014) package in R (2014). Biting midge numbers were log-transformed prior to analysis to control for over-dispersion in the distribution of trap collections.

For each compound, the log-transformed number of biting midges was entered as the dependent variable in the model, with compound release rate entered as a four-level fixed factor (CO_2_-only control set as the reference level of the factor, 0.1 mg h^−1^, 1 mg h^−1^, 10 mg h^−1^). Day (experimental days 1–7) was entered as a random effect. To identify whether there was a significant overall effect of release rate on the number of biting midges collected, likelihood ratio tests were used to compare the residual deviance of models that included and excluded release rate as a factor. Where an overall effect of release rate was found, the significance of differences between number of biting midges caught at 0.1 mg h^−1^, 1 mg h^−1^ and 10 mg h^−1^ compared to the CO_2_-only control was assessed through the model coefficients associated with each release rate. Coefficients with absolute *t*-values greater than 2 were gauged to indicate significantly different (*P* < 0.05) numbers of biting midges caught compared to the CO_2_ only control (Baayen et al. 2008). Results were presented graphically by extracting the model predictions derived from the fixed effects only (release rate), which were subsequently back-transformed onto the original scale. Bootstrapped-confidence intervals (100 simulations) for the predictions were calculated using the boot package in R (Canty and Ripley 2016; Davison and Hinkley 1997), back transformed onto the original measurement scale.

## Results

### Total Number of *Culicoides* Collected and Species Identification

In total, 642,933 biting midges were collected in the control and odor-baited traps. Of these, 99.98% were identified as *C. impunctatus*, with *C. obsoletus senso lato* accounting for the remaining 0.024%.

### Relative Attractiveness of Individual Compounds at Different Release Rates

**Fig. 2:**
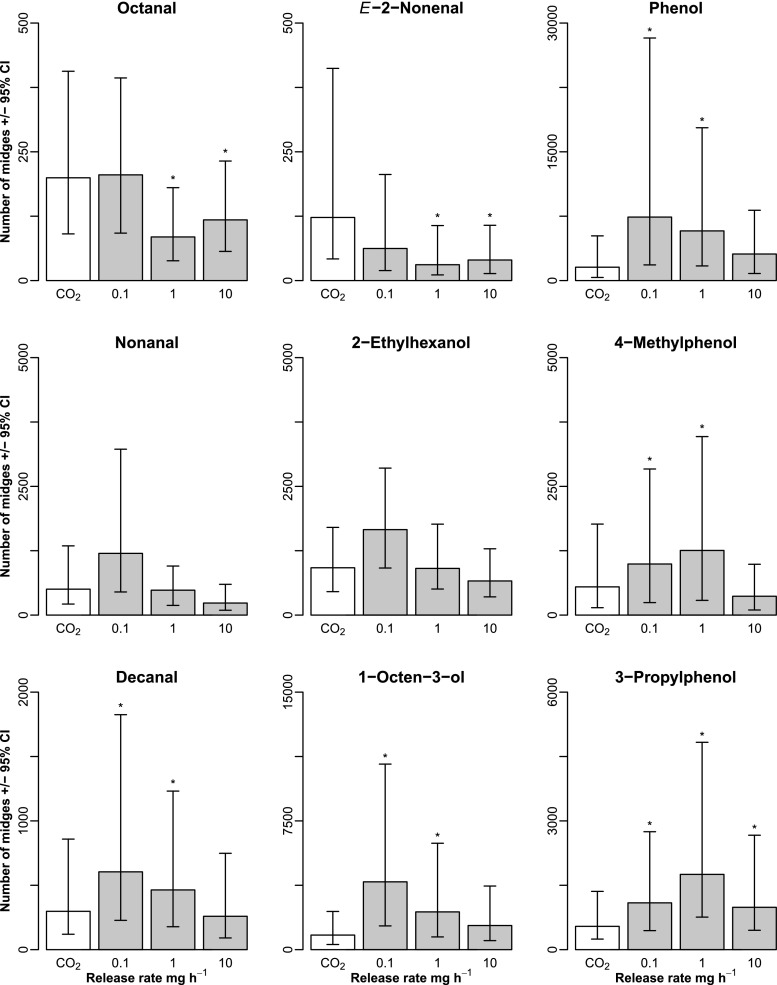
Predicted number of biting midges collected (±95 % confidence intervals. Fixed effects only) for nine cattle-derived odors, when released in combination with carbon dioxide (CO_2_). White bars represent CO_2_-only controls (*N* = 7), and grey bars CO_2_ plus test compound at three different release rates (*N* = 7). Asterisks indicate significant differences (*P* < 0.05) from the CO_2_-only control in the mixed model

A significant effect of the release rate of nonanal on biting midges collected was detected (*χ* ^*2*^ = 8.40, *df* = 3, *P* < 0.05; Fig. 2, middle left). However, none of catches with the different release rates differed significantly from the catch in the trap baited with CO_2_ only. This indicated that a significant difference existed between the number of biting midges collected by traps baited with nonanal released at 0.1 mg h^−1^ and 10 mg h^−1^ (Fig. 2). The release rate of decanal was found to have an impact on the numbers of biting midges collected (*χ* ^*2*^ = 22.1, *df* = 3, *P* < 0.001; Fig. 2, bottom left), and traps baited with decanal released at the two lower rates collected a significantly higher number of biting midges than the CO_2_-only control trap. There was no significant difference in the number of biting midges collected in traps baited with decanal released at 10 mg h^−1^ or those baited with CO_2_ only.

There was a significant overall effect of the octanal release rate on the numbers of biting midges collected (*χ* ^*2*^ = 15.7, *df* = 3, *P* < 0.01; Fig. 2, top left). While there was no significant difference in numbers collected between traps baited with octanal released at 0.1 mg h^−1^ and the CO_2_-only control trap, traps baited with octanal released at 1 mg h^−1^ and 10 mg h^−1^ collected significantly fewer biting midges than the control trap. Similarly, there was an overall effect of the (*E*)-2-nonenal release rate on the numbers of biting midges collected (*χ* ^*2*^ = 8.8, *df* = 3, *P* < 0.05; Fig. 2, top center), and traps baited with (*E*)-2-nonenal at the two highest release rates collected significantly fewer biting midges than the CO_2_-only control trap. However, there was no significant difference in numbers collected between traps baited with the lowest release rate of (*E*)-2-nonenal and the CO_2_ control trap.

The release rate of 2-ethylhexanol had no effect on the number of biting midges collected (*χ* ^*2*^ = 5.6, *df* = 3, *P* > 0.05; Fig. 2, center). In contrast, the release rate of 1-octen-3-ol, had an overall impact on the number of biting midges collected (*χ* ^*2*^ = 16.3, *df* = 3, *P* < 0.001; Fig. 2, bottom center). Traps baited with 1-octen-3-ol released at the two lower rates collected a significantly higher number of biting midges than the CO_2_ control trap, whereas there was no significant difference in numbers collected in the trap baited with 1-octen-3-ol released at 10 mg h^−1^ and the CO_2_-only trap.

The release rate of phenol was found to have an impact on the number of biting midges collected (*χ* ^*2*^ = 7.9, *df* = 3, *P* < 0.05, Fig. 2, top right). Traps baited with phenol released at 0.1 mg h^−1^ and 1 mg h^−1^ collected more biting midges than the CO_2_-only control trap. There was no significant difference in numbers collected between traps releasing phenol at the highest release rate and the control trap. Similarly, the release rate of 4-methylphenol also had an impact on the number of biting midges collected (*χ* ^*2*^ = 16.4, *df* = 3, *P* < 0.01, Fig. 2, middle right). Traps baited with 4-methylphenol released at the two lowest rates collected more biting midges than the CO_2_-only control trap. The number of biting midges collected also varied with release rate of 3-propylphenol (*χ* ^*2*^ = 12.2, *df* = 3, *P* < 0.01; Fig. 2, bottom right), with traps baited with 3-propylphenol at all release rates tested collecting a significantly higher number of biting midges than the CO_2_-only control trap.

## Discussion

Semiochemicals have a demonstrated potential for use in control strategies targeting biting midges (Carpenter et al. 2008a). Building on laboratory results on *C. nubeculosus*, this study showed that some cattle-associated kairomones can be used to enhance attraction of *C. impunctatus* to CO_2_-baited traps in the field. Moreover, some other kairomones reduced trap captures, indicating their potential use as repellents.

Low release rates of decanal, in combination with CO_2_, increased trap captures of *C. impunctatus* when compared to the control CO_2_ trap. This is in agreement with previous studies showing that host-derived aldehydes, either alone or in combination with other host volatiles, play an important role in the attraction of mosquitoes (Syed and Leal 2009; Tchouassi et al. 2013) and tsetse flies (Gikonyo et al. 2003). Tchouassi et al. (2013) also showed that blends of host-derived aldehydes, in combination with CO_2_, were more effective in attracting mosquitoes compared to individual aldehydes. Similar results have been obtained from studies on herbivorous insects showing that behavioral responses to blends of host volatiles often exceed the responses to individual components (Pickett et al. 2010; Webster et al. 2010). An implication of this is that the volatiles may be perceived as non-host cues if detected by the insect individually, outside the context of the blend, but when combined together in a blend they may be perceived as an attractive host stimulus. This may be the case for octanal, nonanal and (*E*)*-*2-nonenal, which, if presented at a lower release rate or in a blend, could be perceived as a host-cue by biting midges. Alternatively, octanal and (*E*)*-*2-nonenal may act as host-derived repellents at higher doses (Jaleta et al. 2016).

The results obtained for 1-octen-3-ol are consistent with those of previous field studies on *C. impunctatus*, *C. nubeculosus* and other biting midge species (Bhasin et al. 2001; Blackwell et al. 1996; Harrup et al. 2012; Kline et al. 1994; Ritchie et al. 1994). 1-Octen-3-ol is a well-characterized mammalian kairomone (Pickett et al. 2010), which is known to attract various hematophagous insects, including tsetse flies (Torr 1990; Vale and Hall 1985) and mosquitoes (Kline et al. 1994; Takken and Kline 1989). As was observed for decanal, *C. impunctatus* were more attracted to traps with a low release rate of racemic 1-octen-3-ol, as previously shown in both laboratory and field studies (Bhasin et al. 2000; Blackwell et al. 1996; Isberg et al. 2016) of *C. impunctatus* and *C. nubeculosus*. While this study analyzed the effect of racemic 1-octen-3-ol on attraction of *C. impunctatus*, other studies on both biting midges and mosquitoes imply that it is the (*R*)*-*enantiomer that is important for attraction (Harrup et al. 2012) and for some species even repellence (Pingxi et al. 2015). Based on the results of Harrup et al. (2012), future field experiments on *C. impunctatus* should validate the effect of (*R*)*-*1-octen-3-ol seen in *C. nubeculosus* and *C. obsoletus.*

Addition of phenol, 4-methylphenol, 3-propylphenol and 2-ethylhexanol, identified in air entrainments of cattle urine (Isberg et al. 2016), to traps baited with CO_2_ increased trap captures of *C. impunctatus*. The results obtained for phenol, as well as 4-methylphenol, agree with those observed for *C. nubeculosus* in the laboratory (Isberg et al. 2016). Phenol, 4-methylphenol and 3-propylphenol, in combination with other host volatiles, have previously been shown to attract *C. impunctatus* (Bhasin et al. 2001) as well as other biting midge species (Cilek et al. 2003; Venter et al. 2011) when compared to an unbaited control traps. Phenolic compounds found in ox urine are also known attractants for tsetse flies (Bursell et al. 1988; Vale et al. 1988) and the zoophilic mosquito *Anopheles quadriannulatus* (Takken and Knols 2007). Unlike tsetse flies that are attracted to blends of phenolic compounds in urine to a level equal to or greater than those with natural urine (Bursell et al. 1988; Torr et al. 1995; Vale et al. 1988), the results from this study suggest that biting midges are attracted to individual phenolic compounds at a similar level to that of natural urine; phenol (0.1 mg h^−1^), 4-methylphenol (0.16–1.41 mg h^−1^) and 3-propylphenol (0.023–0.18 mg h^−1^), and in the case of 3-propylphenol also higher rates than the natural release. In the field experiments presented here, 2-ethylhexanol, when released in combination with CO_2_, did not collect significantly more biting midges than the CO_2_ only control trap, although a larger capture was observed when 2-ethylhexanol was released at 0.1 mg h^−1^. This suggests that lower release rates of 2-ethylhexanol should be tested in future field experiments, an argument also supported by behavioral experiments in the laboratory with *C. nubeculosus* (Isberg et al. 2016).

The attraction of *C. impunctatus* to traps baited with cattle-derived kairomones provides a more diversified set of control and surveillance tools than that currently available (Carpenter et al. 2008a). While semiochemical-based trapping of biting midges is likely to reflect host-seeking populations more accurately than other trapping protocols, the current lures, predominantly 1-octen-3-ol and CO_2_, are not optimal (Carpenter et al. 2008a; Carpenter et al. 2008b; Gerry et al. 2009; Harrup et al. 2012; Viennet et al. 2011). Future studies will have to assess whether the compounds originally identified to modulate the behavior of *C. nubeculosus* in the laboratory, and then of *C. impunctatus* in the field, also attract other *Culicoides* species. Further optimization of attractive lure(s) could involve analyzing the effect of blends, including host-derived aldehydes or phenolic compounds, on biting midge behavior. The findings that some host-derived volatiles can inhibit the host seeking behavior of *Culicoides* biting midges (Isberg et al. 2016; this study) merit further investigation. These repellents might be an innovative way to disrupt the host-seeking behavior of biting midges, similar to what has been shown for tsetse flies (Gikonyo et al. 2002) and malaria mosquitoes (Jaleta et al. 2016).
